# Commercial Vaccines Do Not Confer Protection against Two Genogroups of *Piscirickettsia salmonis*, LF-89 and EM-90, in Atlantic Salmon

**DOI:** 10.3390/biology11070993

**Published:** 2022-06-30

**Authors:** Carolina Figueroa, Débora Torrealba, Byron Morales-Lange, Luis Mercado, Brian Dixon, Pablo Conejeros, Gabriela Silva, Carlos Soto, José A. Gallardo

**Affiliations:** 1 Laboratorio de Genética y Genómica Aplicada, Escuela de Ciencias del Mar, Pontificia Universidad Católica de Valparaíso, Avenida Universidad 330, Valparaíso 2373223, Chile; cfigueroa.tm@gmail.com (C.F.); debora.torrealba@pucv.cl (D.T.); 2 Grupo de Marcadores Inmunológicos en Organismos Acuáticos, Instituto de Biología, Pontificia Universidad Católica de Valparaíso, Avenida Universidad 330, Valparaíso 2373223, Chile; byron.maximiliano.morales.lange@nmbu.no (B.M.-L.); luis.mercado@pucv.cl (L.M.); 3 Department of Biology, Faculty of Science, University of Waterloo, 200 University Avenue West Waterloo, Waterloo, ON N2L3G1, Canada; bdixon@uwaterloo.ca; 4 Centro de Investigación y Gestión de Recursos Naturales (CIGREN), Instituto de Biología, Facultad de Ciencias, Universidad de Valparaíso, Avenida Gran Bretaña 1111, Valparaíso 2360102, Chile; pablo.conejeros@uv.cl; 5 Salmones Camanchaca, Diego Portales 2000, Puerto Montt 5503642, Chile; gabriela.silva@bmkgenetics.com (G.S.); csotov@camanchaca.cl (C.S.)

**Keywords:** pentavalent vaccine, bacterin vaccine, live attenuated vaccine, monovalent vaccine, *Piscirickettsiosis*, *Salmo salar*, cohabitation, sea lice, vaccine efficacy

## Abstract

**Simple Summary:**

Vaccination represents one of the most relevant strategies to prevent and control infectious diseases in aquaculture. However, vaccines have failed to control and prevent *Piscirickettsia salmonis*, a bacterium that causes large economic losses to the industry. Therefore, we evaluated the performance of two commercial vaccines in Atlantic salmon through a cohabitation challenge (healthy fish were challenged by cohabitation with infected fish) of the two most prevalent and ubiquitous *Piscirickettsia* genetic variants in Chile. We found no evidence that vaccines confer protection against the LF-89 or EM-90 genogroups in Atlantic salmon.

**Abstract:**

In Atlantic salmon, vaccines have failed to control and prevent *Piscirickettsiosis*, for reasons that remain elusive. In this study, we report the efficacy of two commercial vaccines developed with the *Piscirickettsia salmonis* isolates AL100005 and AL 20542 against another two genogroups which are considered highly and ubiquitously prevalent in Chile: LF-89 and EM-90. Two cohabitation trials were performed to mimic field conditions and vaccine performance: (1) post-smolt fish were challenged with a single infection of LF-89, (2) adults were coinfected with EM-90, and a low level coinfection of sea lice. In the first trial, the vaccine delayed smolt mortalities by two days; however, unvaccinated and vaccinated fish did not show significant differences in survival (unvaccinated: 60.3%, vaccinated: 56.7%; *p* = 0.28). In the second trial, mortality started three days later for vaccinated fish than unvaccinated fish. However, unvaccinated and vaccinated fish did not show significant differences in survival (unvaccinated: 64.6%, vaccinated: 60.2%, *p* = 0.58). Thus, we found no evidence that the evaluated vaccines confer effective protection against the genogroups LF-89 and EM-90 of *P. salmonis* with estimated relative survival proportions (RPSs) of −9% and −12%, respectively. More studies are necessary to evaluate whether pathogen heterogeneity is a key determinant of the lack of vaccine efficacy against *P. salmonis*.

## 1. Introduction

*Piscirickettsia salmonis* is a major concern for the Chilean salmon industry, causing economic losses of USD 700 million per year [1,2]. *Piscirickettsiosis* is an exceptionally contagious disease, with mortalities of over 50% in regions of Chile where prevalence is high [3]. While Chile, the second-largest global producer of salmon, is by far the country most affected by this disease, it also affects the other main salmon producing countries, namely Norway, Canada, and Scotland [4,5,6,7].

Vaccination has been widely used as a control strategy to prevent *Piscirickettsiosis* [8], but unfortunately, all the vaccines developed in the last 20 years have failed to protect Atlantic salmon against *P. salmonis* [1]. Some intrinsic and extrinsic factors that may explain why commercial vaccines do not provide protection against *P. salmonis* are: (1) coinfection with sea lice, which can override the protective effects of vaccines [9,10]; (2) host genetic variation, partially protecting some hosts while leaving others unprotected [9,10]; and (3) ineffectiveness in stimulating cellular immunity, which is a key element to protecting against *P. salmonis* because this bacterium can survive inside the host cells. Likewise, other underlying causes may lead to low vaccine efficacy, such as pathogen genetic variation or a poor match between the vaccine and the circulating strain.

Since outbreaks of *Piscirickettsiosis* in Chile are mainly caused by two genetic strains of *P. salmonis*, it has been suggested that this heterogeneity should be considered in vaccine development [11,12]. The reported efficacy of a commercial vaccine would be expected to be low when testing against bacterial strains with low virulence and/or a reduced prevalence in the field [11]. In Chile, two strains—called LF-89 and EM-90—are considered highly and ubiquitously prevalent [13]. These strains show distinct laboratory growth conditions [13] and have major differences in virulence-associated secretion systems and transcriptional profiles [14], resulting in different levels of infectivity [15]. For example, it has been shown that the EM-90-like strain is more aggressive than LF-89, inducing higher cumulative mortalities (EM-90 = 95%; LF-89 = 82%) with a shorter time to death (EM-90 = 42 days; LF-89 = 46 days) in non-vaccinated post-smolts when evaluated by a cohabitation challenge [16,17]. Contrary to the hypothesis of heterogeneity, an experimental vaccine developed from strain EM-90 failed to protect against that same strain [18,19].

In this study, we tested the efficacy of two commercial vaccines against the two most prevalent Chilean genogroups of *P. salmonis*, LF-89, and EM-90. The first vaccine was a pentavalent bacterin injectable vaccine (AL100005 isolate), and the second was a combination of the pentavalent bacterin vaccine with a live attenuated injectable vaccine (AL 20542 isolate). The cohabitation challenges were carried out with Atlantic salmon (*Salmo salar*) that were successfully adapted to salt water to best imitate the natural conditions of bacterial infection. In the first trial, LF-89 was evaluated in post-smolt fish given a single infection of *P. salmonis*, while in the second trial, EM-90-like was evaluated with adult fish in a challenge that included a very low coinfection with sea lice (*C. rogercresseyi)*, to once again better emulate field conditions.

## 2. Materials and Methods

### 2.1. Ethics Statement

This work was carried out under the Canadian Council on Animal Care guidance for the care and use of experimental animals. The protocol was approved by the Bioethics Committee of the Pontificia Universidad Católica de Valparaíso and the Comisión Nacional de Investigación Científica y Tecnológica de Chile (FONDECYT No. 1140772). Animals were fed daily ad libitum with a commercial diet. To reduce stress during handling, vaccination was performed on fish sedated with AQUI-S (50% Isoeugenol, 17 mL/100 L water). Fish were euthanized by an overdose of anesthesia (AQUI-S, 50 mL/100 L).

### 2.2. Commercial Vaccines

The commercial vaccines, hereafter “the vaccine”, used in this study were a pentavalent bacterin vaccine (ALPHA JECT 5-1^®^; PharmaQ AS, Overhalla, Norway) with antigens against *P. salmonis*, *Vibrio ordalii*, *Aeromonas salmonicida*, IPNV (Infectious Pancreatic Necrosis Virus) and ISAV (Infectious Salmon Anemia Virus) and a monovalent live attenuated vaccine against *P. salmonis* (ALPHA JECT LiVac^®^ SRS; PharmaQ AS, Overhalla, Norway). This pentavalent vaccine is used by 43% of Chilean farmers (3821 vaccination events over 8884 events in the freshwater phase of production) and thus is the most commonly used vaccine, while the live attenuated vaccine is fifth in terms of usage at 6.8% (608/8884) [8]. The component of these vaccines included for prevention of *Piscirickettsiosis* is the *P. salmonis* AL 10005 strain (pentavalent vaccine) and *P. salmonis* AL 20542 (live attenuated vaccine), respectively. The pentavalent vaccine was used in trial 1, and both vaccines (pentavalent and live attenuated) were given simultaneously in trial 2 as recommended by the manufacturer. No fish was challenged before first completing the time period required for protection indicated by the manufacturer (ALPHA JECT 5-1 = 600 UTA or 46.15 days at 13 °C; ALPHA JECT LiVac = 456 UTA or 35.07 days at 13 °C).

### 2.3. Challenge with LF-89 Genogroup (Trial 1)

A total of 4987 individually pit-tagged smolt Atlantic salmon were provided in 2017 by Salmones Camanchaca (Puerto Montt, Chile). Fish were transferred to the Neosalmon experimental station (Puerto Montt, Chile) for the cohabitation challenge (Table 1). Smolts were received into a salinity of 6–8 ppt, which was gradually increased over 14 days to 32 ppt. In total, 1002 of the fish previously immunized with the vaccine using the normal production schedule were used as vaccinated fish (342 ± 55 g), while 1062 fish that had been previously injected with Phosphate-Buffered Saline (PBS) were used as unvaccinated fish (314 ± 61 g). The remaining fish were used as Trojan shedders (152 ± 38 g).

Vaccinated and unvaccinated fish were distributed into four tanks: two tanks of 15 m^3^ for the cohabitation challenges and two tanks of 5 m^3^ for the control without infection. All fish were acclimatized to the experimental conditions (salinity of 32 ppt and a temperature of 15 ± 1 °C) and tanks for at least 15 days prior to the challenge. Further, a health check by RT-PCR was performed by ADL Diagnostic Chile Company (Puerto Montt, Chile) to verify that the fish were free of viral (ISAV and IPNV) and bacterial pathogens (*Vibrio ordalii*, *Flavobacterium psychrophilum*, *P. salmonis*, and *Renibacterium salmoninarum*). RT-PCR was performed following SERNAPESCA regulations [3]. The cohabitation tanks were challenged by adding Trojan shedders (Table 1, Figure S1A) which had been previously injected with a median lethal dose (LD_50_ of 1 × 10^−2^ TCID/mL:TCID: median tissue culture infective dose) of the LF-89 genogroup (isolate PM-38986) provided by ADL Diagnostic Chile (Puerto Montt, Chile). The experiment was conducted for 43 days after the *P. salmonis* injection of Trojans (Figure S1A). The LD_50_ used in Trojans was previously determined on 800 fish immunized with the vaccine, which were equally distributed in four treatments and two tanks of 1000 L per treatment. Treatment 1 involved injection with 1 × 10^−2^ TCID/mL, treatment 2 involved injection with 1 × 10^−3^ TCID/mL, treatment 3 involved injection with 1 × 10^−4^ TCID/mL, and treatment 4 involved injection with PBS. Fish were monitored daily for 30 days, and mortalities were recorded.

### 2.4. Challenge with EM-90-LIKE Genogroup and Coinfection with sea Lice (Trial 2)

A total of 442 individually pit-tagged adult fish were provided in 2019 by Salmones Camanchaca (Puerto Montt, Chile) and transferred to the Aquadvice experimental station (Puerto Montt, Chile) for the cohabitation challenge (Table 1). In total, 121 of the fish, which had been previously immunized with the vaccine using the normal production schedule, were used as vaccinated fish (1294 ± 326 g), while 138 that had been previously injected with PBS were used as unvaccinated fish (1228 ± 345 g). The remaining fish were used as Trojan shedders (1308 ± 337 g).

Vaccinated and unvaccinated fish were distributed into three tanks of 11 m^3^: two tanks for the cohabitation challenges and one tank for the control without infection. All fish were acclimatized to the experimental conditions (salinity of 32 ppt and a temperature of 15 ± 1 °C) for at least 15 days prior to the challenge. Further, a health check by RT-PCR was performed by ADL Diagnostic Chile Company (Puerto Montt, Chile) to verify that the fish were free of viral (ISAV and IPNV) and bacterial pathogens (*Vibrio ordalii*, *Flavobacterium psychrophilum*, *P. salmonis*, and *Renibacterium salmoninarum*). The cohabitation tanks were challenged by adding Trojan shedders (Table 1, Figure S1B) which had been previously injected with a median lethal dose (LD_50_) of 1 × 10^−3.5^ TCID/mL of the EM-90 genogroup (isolate PS03-04) provided by Fraunhofer Chile (Santiago, Chile). Seven days after the Trojan fish were challenged with *P. salmonis*, all fish (cohabitant, Trojan and control) were infested with *C. rogercresseyi* copepodids. The coinfection procedure was established based on our previous studies [20], but in this case a low infection rate was applied to mimic the natural infection rates normally seen in field conditions [21]. Infections with sea lice were performed by adding 20 copepodites per fish to each control and coinfection tank. Copepodites were collected from egg-bearing females reared in the laboratory and confirmed to be pathogen-free (*P. salmonis*, *R. salmoninarum*, *IPNV*, and *ISAV*) by RT-PCR. After the addition of parasites, water flow was stopped for a period of 8 h, and tanks were covered to decrease light intensity, which favors the successful settlement of sea lice on fish [20]. Parasite counts were performed a week after the infestation for nine fish per tank. The challenge lasted 60 days after the Trojans’ infection with *P. salmonis*.

The LD_50_ used in Trojans was previously determined on 330 immunized fish, which were equally distributed in five treatments and two tanks of 720 L per treatment. Treatment 1 involved injection with 1 × 10^−1.5^ TCID/mL, treatment 2 involved injection with 1 × 10^−2.5^ TCID/mL, treatment 3 involved injection with 1 × 10^−3.5^ TCID/mL, treatment 4 involved injection with 1 × 10^−4.5^ TCID/mL, and treatment 5 involved injection with PBS. Fish were monitored daily for 30 days, and mortalities were recorded (Figure S1B).

### 2.5. Necropsy Analysis

Macroscopic lesions from 10 controls and 10 cohabitant fish in each trial were analyzed. Two different veterinarians who were blinded to the treatments studied fresh samples from trials 1 or 2. In the challenge with LF-89 genogroup, macroscopic lesions in the liver were evaluated at 21 days post-infection, where vacuolar degeneration, hepatitis, and hepatocyte atrophy were described according to their presence or absence. Additionally, 47 vaccinated and unvaccinated fish from cohabitation and control tanks were analyzed by immunohistochemistry to detect the presence or absence of *P. salmonis* in the liver both 21 days after the challenge and at the end of the experiment. For the EM-90 genogroup experiment, pathological signs were evaluated only at the end of the challenge; this analysis included the presence or absence of nodules in the liver, congestive liver, and hepatomegaly.

### 2.6. ELISA

An indirect Enzyme-Linked Immunosorbent Assay (ELISA) was performed in serum samples from the first trial only—the fish challenged with the LF-89 genogroup. Secretion levels of total immunoglobulin (Igs), antigen-specific immunoglobulins against *P. salmonis* (spIgs), tumor necrosis factor-alpha (Tnfα) and interferon-gamma (Ifnγ) were measured following the protocol of Morales-Lange et al. [22]. Briefly, the total protein concentration of each sample was determined by the BCA (Bicinchoninic acid) method (Pierce, Thermo Fisher, Waltham, MA, USA) according to the supplier’s instructions. Then, each sample was diluted in carbonate buffer (60 mM NaHCO_3_, pH 9.6), seeded in duplicate at 50 ng µL^−1^ (100 µL) in a Maxisorp plate (Nunc, Thermo Fisher Scientific, Waltham, USA) and incubated overnight at 4 °C. After that, the plates were blocked with 200 µL per well of 1% Bovine Serum Albumin (BSA) for 2 h at 37 °C, and later the primary antibodies (Table S1 and Figure S2) [23] were added for 90 min at 37 °C. Next, a secondary antibody—HRP (Thermo Fisher)—was added for a 60 min incubation at 37 °C at a 1:7000 dilution. Finally, 100 µL per well of chromogen substrate 3,30,5,50-tetramethylbenzidine (TMB) single solution (Invitrogen, Carlsbad, CA, USA) was added and the plates were incubated for 30 min at room temperature. The reaction was stopped with 50 µL of 1 N sulfuric acid and read at 450 nm on a VERSAmax microplate reader (Molecular Devices, San Jose, CA, USA). For the detection of spIg, 50 ng µL^−1^ of total protein extract from *P. salmonis* [24] were seeded per well in a Maxisorp plate (diluted in 100 µL of carbonate buffer) and incubated overnight at 4 °C. After blocking with 1% BSA (200 µL per well), each fish serum sample was incubated in duplicate at a total Igs concentration of 50 ng µL^−1^ for 90 min at 37 °C. After that, the ELISA protocol described above was followed.

### 2.7. Statistical Analysis

The mortality was recorded, and data were represented using Kaplan–Meier survival curves [25]. The protection elicited by vaccines was determined by comparing the survival percentage of vaccinated and unvaccinated groups using a Log-rank test. Further, the Relative Proportion Survival (RPS) was calculated as

RPS (%) = (1−A/B) ∗ 100

where A and B are the mortalities at the end of challenges in vaccinated and unvaccinated fish, respectively.

Additionally, differences in the pathological symptoms of *P. salmonis* infection between different treatments were analyzed using a non-parametric Chi-square test. Finally, significant differences in ELISA tests were compared using the Student’s two-tailed *t*-test, *p* < 0.05. All statistical analyses were performed using R Core Team (RStudio, Vienna, Austria). Graphs were designed with GraphPad Prism 8.0 software (GraphPad Software, San Diego, CA, USA).

## 3. Results

### 3.1. Vaccine Efficacy against the LF-89 Genogroup

**Figure 1:**
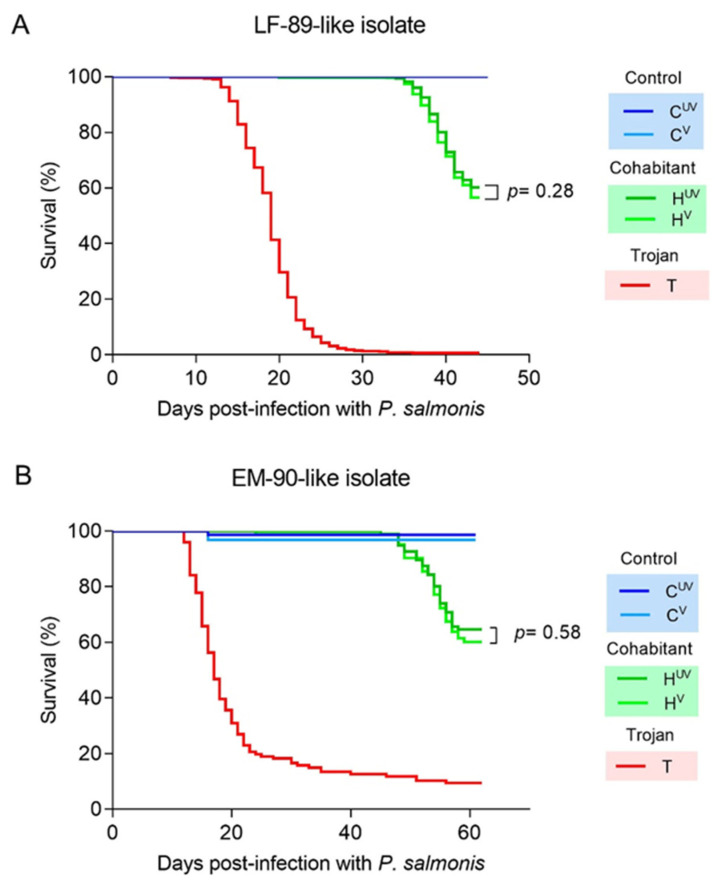
Survival curves: (**A**) Single infection of Atlantic salmon post-smolt with the *P. salmonis* LF-89 genogroup. (**B**) Coinfection of Atlantic salmon adults with the *P. salmonis* EM-90 genogroup and the sea louse *C. rogercresseyi*. Fish from the first trial were immunized with pentavalent injectable vaccine, and fish from the second trial with pentavalent injectable plus monovalent live attenuated injectable. Abbreviations: C^UV^: control unvaccinated; C^V^: control vaccinated; H^UV^: cohabitant unvaccinated; H^V^: cohabitant vaccinated; T: Trojan.

No mortality was recorded in the non-infected control fish. However, the cohabitation challenge with the LF-89 strain resulted in high mortality in the experimental fish. There was no evidence that the pentavalent vaccine generated effective protection against the LF-89 genogroup. The vaccine delayed mortalities by two days (H^UV^: 34 dpi and H^V^: 36 dpi), but both unvaccinated and vaccinated fish showed similar survival during and at the end of the challenges (H^UV^: 60.3% and H^V^: 56.7%, Figure 1A). Therefore, the survival test did not reveal significant differences between vaccinated and unvaccinated treatments (*p* = 0.28).

**Figure 2:**
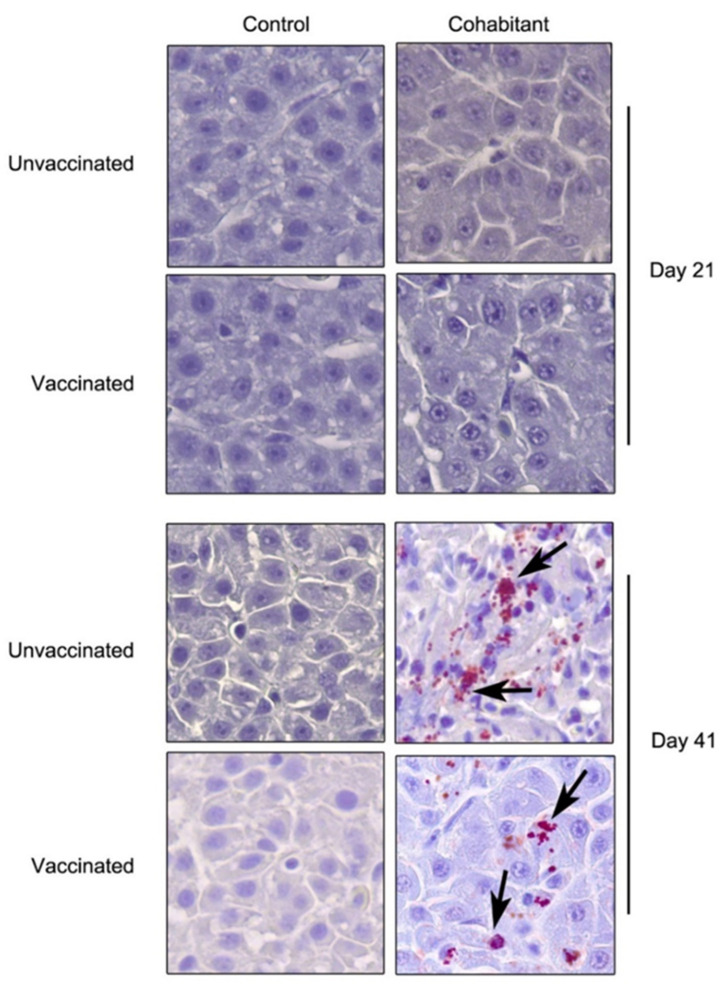
Presence of LF-89 genogroup of *P. salmonis* (black arrows) in liver samples of Atlantic salmon. *Piscirickettsiosis* was detected in 11 out of 47 fish analyzed by immunohistochemistry—magnification 63X.

Dead fish and large numbers of vaccinated and unvaccinated live fish at the end of the challenge showed multiple hemorrhagic ulcers on the skin typical of a severe *P. salmonis* infection. *P. salmonis* infection was also evident in the liver of both vaccinated and unvaccinated fish at the end of the challenge, but not at 21 days after infection (Figure 2). On the other hand, vaccination increased the presence of hepatocyte atrophy in comparison with unvaccinated fish in the control treatment at 21 days post-infection (Table 2). A similar trend was observed in the cohabitant fish, but without significant differences (Table 2). Once the challenge was over, the fish were evaluated for most common salmon diseases, revealing the appearance of secondary infections of *Piscine orthoreovirus* (Figure S3) and *Tenacibaculum dicentrarchi* in some animals.

**Figure 3:**
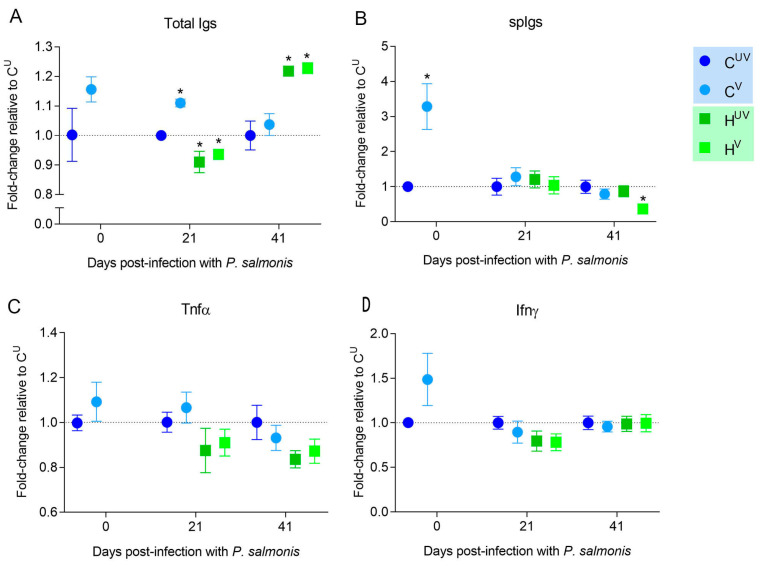
Protein levels by ELISA: Secretion of total Igs (**A**), antigen specific Igs (**B**), tumor necrosis factor alpha (Tnfα) (**C**), and interferon gamma (Ifnγ) (**D**) in serum samples from Atlantic salmon measured by ELISA after a challenge with *P. salmonis* in the first trial (single infection of the LF-89 genogroup). Fish immunized with pentavalent injectable vaccine. Data represent the mean ± SEM (*n* = 10). Significant differences compared to C^UV^ by Student t-test two-tailed (* = *p* < 0.05). Abbreviations: C^UV^: control unvaccinated; C^V^: control vaccinated; H^UV^: cohabitant unvaccinated; H^V^: cohabitant vaccinated.

Serum samples showed a significant increase of total Igs at 21 days post-infection (Figure 3A) in the control group of vaccinated fish (C^V^). However, at the same sampling time, both unvaccinated and vaccinated experimental fish showed a decrease in total Igs levels. This trend was reversed at 41 dpi, since both groups (H^UV^ and H^V^) significantly increased their levels of total Igs. On the other hand, when specific immunoglobulins against *P. salmonis* were measured (Figure 3B), an increase was detected in C^V^ even before the challenge with *P. salmonis*. Nevertheless, after 41 days post-infection, H^V^ group showed lower titers of *P. salmonis* spIgs than the other groups. Finally, the evaluation of Tnfα and Ifnγ secretion did not show significant changes between treatments (Figure 3C,D).

### 3.2. Vaccine Efficacy against the EM-90 Genogroup with Low Sea Lice Coinfection

In the second trial, adult fish were coinfected with sea lice to mimic natural conditions in the field. Seven days after sea lice infestation, the prevalence of sea lice was 100% in treatment and control tanks, with no significant differences in the abundance of the parasites between tanks (Tank 1 = 10.4 ± 4.0; Tank 2 = 11.7 ± 3.0; Control tank = 9.7 ± 6.6). The vaccine (pentavalent injectable + monovalent live attenuated injectable) was not able to protect against the EM-90 genogroup (Survival percent: H^V^: 60.2% and H^UV^: 64.6%; Figure 1B; *p* = 0.58) in cohabitant fish with low-level sea lice infection. However, a small effect of delayed mortalities was observed; for example, steady mortality started three days later for vaccinated fish compared with unvaccinated fish (H^V^: 48 dpi and H^UV^: 45 dpi). The control tank infected only with sea lice experienced very low mortality, with one in the unvaccinated fish (C^UV^) and two in the vaccinated fish (C^V^).

Vaccinated and unvaccinated mortalities showed hemorrhagic ulcers on the skin typical of a severe *P. salmonis* infection. Further, when we compared cohabitant and control fish at the end of the challenges, infection with *P. salmonis* was evident in the cohabitant fish through the three evaluated pathological signs: nodules in liver, congestive liver, and hepatomegaly (Table 3). However, we did not find differences between vaccinated and unvaccinated fish in cohabitant fish (Table 3). For instance, in the cohabitant treatment, nine unvaccinated fish presented a congestive liver, compared to 10 vaccinated fish showing that symptom. Similar patterns were found for nodules in the liver and hepatomegaly.

## 4. Discussion

Vaccination is one of the most used strategies to prevent and control diseases in aquaculture [26,27]. However, vaccines have failed to control and prevent *Piscirickettsiosis*, for reasons that remain elusive [1,27,28,29]. This manuscript evaluated whether the heterogeneity of *P. salmonis* could explain the low vaccine efficacy of a commercial vaccine whose active principle is a bacterin developed using the *P. salmonis* AL 10005 strain and a live attenuated vaccine developed with AL 20542 isolates. To achieve this, we evaluated the vaccine efficacy using the two most prevalent and ubiquitous genetic variants of *P. salmonis* in Chile. Challenges were designed to mimic the natural conditions of infection; thus, LF-89 was evaluated with post-smolt fish in a single infection of *P. salmonis*, and EM-90 was evaluated with adult fish in a challenge that included a very low coinfection with the sea louse *C. rogercresseyi*. In this study, we found no evidence that vaccines developed with the *P. salmonis* AL 10005 or AL 20542 isolates confer protection against infection caused by the LF-89 or EM-90 genogroups in Atlantic salmon.

The absent or low level of protection provided by the commercial vaccines against infection caused by *P. salmonis* in the field could be related to the use of a model for the evaluation of protection in the product development vaccination trials that is not reproducing real field conditions. For example, the route of infection has been proposed as a relevant factor in the performance of a vaccine. Here, we selected a cohabitation model of challenges, because this best mimics the natural infection route [30]. On the other hand, several studies evaluating vaccine efficacy against *P. salmonis* have been performed by intraperitoneal injection [31,32,33,34]. Intraperitoneal injection is preferred because it is a synchronized and effective infection route that shortens the time to produce disease symptoms, decreasing the cost of trials [18,19]. Vaccine efficacy has previously been found to be affected by the route of infection for furunculosis [35] but not for *Piscirickettsiosis* in Atlantic salmon [19].

Moreover, coinfection with other pathogens such as sea lice is usually not considered in the evaluation of *P. salmonis* vaccine efficacy in laboratory-controlled conditions. We consider that this overestimates the true ability of vaccines to control *Piscirickettsiosis* for three reasons: first, sea lice are highly prevalent in the ocean; second, the long culture times in the sea ensure that fish will be infected not once but several times by this parasite; third, it has been shown that sea lice infection can override the protective effects of vaccination [9]. We observed no pathological signs associated with *P. salmonis* in the control tank, and mortality was significantly lower in the control tanks (less than 2%; 3 of 137 fish) than in the coinfection treatment animals (36–40%). Because we did not observe differences in mortality or pathological signs between vaccinated and unvaccinated adult fish in the cohabitation experiment we predict that the evaluated vaccine will not protect fish in the field.

The immune mechanisms involved in vaccine protection against *P. salmonis* are poorly understood. In this research, the vaccine was able to induce an increase of spIgs in vaccinated fish. However, this occurred before the challenge with *P. salmonis*. After the challenge, cohabiting fish showed increases only in total Igs (41 dpi) and even a decrease of spIgs against *P. salmonis* by 41 dpi, perhaps due to B cell depletion. Apparently, the vaccine is not able to activate components of acquired immunity such as specific antibodies or cytokines associated with T_H_1 profiles (Tnfα and Ifnγ) once fish face *P. salmonis* infection, perhaps because *P. salmonis* is an intracellular parasite that requires a T_H_2 response that inhibits T_H_1 responses. This suggests that the vaccine could act as an immunostimulant for the adaptive response at early time points, but not as a vaccine that induces future specific secondary responses. It has already been reported that vaccines may induce weaker or shorter-lived immunity in fish, mainly due to the low immunogenicity of the antigens used or because they cannot modulate the antigen presentation processes effectively during the different stages of immunity [36]. Therefore, the protective mechanism that *P. salmonis* vaccines might have in the field [8] needs to be clarified.

In Chile, the Agricultural and Livestock Service of Chile (SAG) authorized *P. salmonis* vaccines that meet a minimum protection of ≥70% RPS in experimental trials to be marketed. However, there is little evidence of their effectiveness under field conditions [8]. In this study, the minimum protection of ≥70% RPS was not reproduced either against *P. salmonis* LF-89 genogroup or in the EM-90 genogroup. Unfortunately, neither the pharmaceutical companies nor the SAG (Agricultural and Livestock Service) publicly release the results of efficacy studies that authorize the marketing of vaccines in Chile. This prevented us from comparing our results with the efficacy studies carried out by pharmaceutical companies. Vaccine efficacy studies must be public and must consider both the genetic heterogeneity of the host and the pathogen’s heterogeneity. In fact, we do not know whether pathogen heterogeneity was considered or if the most vulnerable populations of fish were included when the efficacy of the *Piscirickettsiosis* vaccine was evaluated by the SAG, as is recommended by the World Organization for Animal Health (OIE).

## 5. Conclusions

Commercial vaccines against *P. salmonis* have failed to reduce mortality or prevent outbreaks in field conditions. In this study, we found no evidence that commercial vaccines confer protection in Atlantic salmon against the LF-89 or EM-90 genogroups of *P. salmonis*. We have provided insights into the heterogeneity of *P. salmonis* that could explain the low efficacy of commercial vaccines whose active agent is different to the two most prevalent and ubiquitous naturally occurring genetic variants of *P. salmonis* in Chile.

## Figures and Tables

**Table 1:** Number and proportion of Atlantic salmon used per group and treatment for the first and second trials. In the first trial, post-smolt fish were challenged with the LF-89 genogroup of *P. salmonis*, while in the second trial, adult fish were challenged with the EM-90 genogroup of *P. salmonis* and with the sea lice *C. rogercresseyi*.

Group	Treatments	First Trial	Second Trial
Cohabitant	Vaccinated (H^V^)	496	83
	Unvaccinated (H^UV^)	335	96
	Total cohabitant (H)	831	179
	**H^UV^/H**	**42%**	**53%**
Trojan	Total Trojans (T)	2903	183
	**T/(H + T)**	**77%**	**41%**
Control	Vaccinated (C^V^)	506	38
	Unvaccinated (C^UV^)	727	42
	Total control (C)	1233	80
	**Total fish (H + T + C)**	**4987**	**442**

**Table 2:** Pathological signs in Atlantic salmon challenged with the LF-89 genogroup of *P. salmonis* at day 21 post-infection in cohabitant and control groups. Differences between vaccinated and unvaccinated fish were evaluated with a Chi-squared statistical test (* = *p* < 0.05). Abbreviations: UV: unvaccinated fish and V: vaccinated fish.

Group	Pathological Signs	Presence of Pathological Signs	Treatment		Proportion		Chi-Square Test	
			UV	V	UV	V	*X* ^2^	*p*-Value
Cohabitant	Vacuolar	No	2	4	0.2	0.4	0.24	0.63
	degeneration	Yes	8	6	0.8	0.6		
		Total	10	10				
	Hepatitis	No	9	9	0.9	0.9	0	1
		Yes	1	1	0.1	0.1		
		Total	10	10				
	Hepatocyte	No	8	4	0.8	0.4	1.88	0.17
	atrophy	Yes	2	6	0.2	0.6		
		Total	10	10				
Control	Vacuolar	No	1	3	0.1	0.3	0.31	0.58
	degeneration	Yes	9	7	0.9	0.7		
		Total	10	10				
	Hepatitis	No	8	7	0.8	0.7	0	1
		Yes	2	3	0.2	0.3		
		Total	10	10				
	Hepatocyte	No	10	5	1	0.5	4.27	<0.05 *
	atrophy	Yes	0	5	0	0.5		
		Total	10	10				

**Table 3:** Pathological signs in Atlantic salmon challenged with the EM-90 genogroup of *P. salmonis* and infestation with *C. rogercresseyi* at day 47–51 post-infection in cohabitant and control groups. Differences between vaccinated and unvaccinated fish were evaluated with a Chi-squared statistical test. Abbreviations: UV: unvaccinated fish and V: vaccinated fish.

Group	Pathological Signs	Presence of Pathological Signs	Treatment		Proportion		Chi-Square Test	
			UV	V	UV	V	*X* ^2^	*p*-Value
Cohabitant	Nodules in	No	0	0	0	0	0	1
	liver	Yes	10	10	1	1		
		Total	10	10				
	Congestive	No	1	0	0.1	0	0.02	0.96
	liver	Yes	9	10	0.9	1		
		Total	10	10				
	Hepatomegaly	No	0	0	0	0	0	1
		Yes	10	10	1	1		
		Total	10	10				
Control	Nodules in	No	10	10	1	1	0	1
	liver	Yes	0	0	0	0		
		Total	10	10				
	Congestive	No	10	9	1	0.9	0	1
	liver	Yes	0	1	0	0.1		
		Total	10	10				
	Hepatomegaly	No	10	9	1	0.9	0	1
		Yes	0	1	0	0.1		
		Total	10	10				

## Data Availability

Data supporting the reported results can be found in this GitHub repository: https://github.com/GenomicsLaboratory/ReproducibleResearch (accessed on 12 May 2022).

## Supplementary Materials

The following supporting information can be downloaded at: https://www.mdpi.com/article/10.3390/biology11070993/s1, Figure S1: Experimental pipeline (**A**) Single infection of Atlantic salmon post-smolt with the P. salmonis LF 89-like genogroup. (**B**) Coinfection of Atlantic salmon adults with the *P. salmonis* EM-90-like genogroup and the sea louse *C. rogercresseyi*. Table S1: Primary polyclonal antibodies used in ELISA analysis; Figure S2: Validation of antibodies against total serum immunoglobulins (Igs) of *Salmo salar*: (**A**) Indirect ELISA calibration curve between total serum Igs concentration of *S. salar* (ng µL^−1^) and optical density at 450 nm; (**B**) Western blot. Antibodies were produced in mice using total serum Igs from Atlantic salmon as antigen. The antigen was obtained by the caprylic acid technique for immunoglobulin purification [23]; Figure S3: Presence of *Piscine orthoreovirus* (black arrows) in heart samples of Atlantic salmon from the first trial at day 41 post infection with LF-89 genogroup of *P. salmonis*. The virus was detected in 7 out of 17 fish analyzed by immunohistochemistry—magnification 63X.
