# Supplementary material for: Commercial Vaccines Do Not Confer Protection against Two Genogroups of Piscirickettsia salmonis, LF-89 and EM-90, in Atlantic Salmon
Source: Biology (Basel). 2022 Jun 30;11(7):993. doi: 10.3390/biology11070993 (PMC9312220; doi:10.3390/biology11070993)
Supplement: Supplementary file 1 [file biology-11-00993-s001.zip › biology-1748026-supplementary.pdf]

Supplementary Materials

Commercial vaccines do not confer protection against two genetic strains of *Piscirickettsia salmonis*, LF-89-like and EM-90-like, in Atlantic salmon.

Carolina Figueroa, Débora Torrealba, Byron Morales-Lange, Luis Mercado, Brian Dixon, Pablo Conejeros, Gabriela Silva, Carlos Soto, José A. Gallardo*

*** Correspondence:** jose.gallardo@pucv.cl

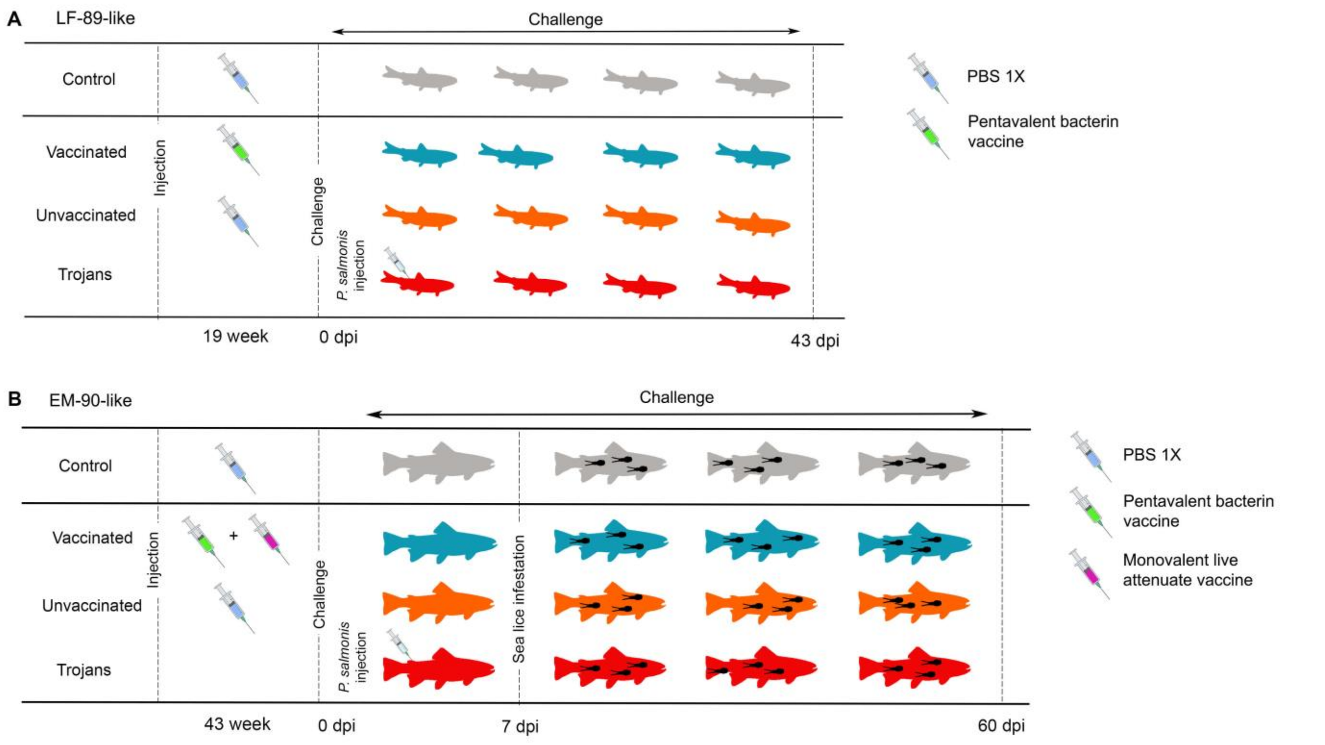

Figure S1. Experimental pipeline (A) Single infection of Atlantic salmon post-smolt with the *P. salmonis* LF-89-like genogroup. (B) Coinfection of Atlantic salmon adults with the *P. salmonis* EM-90-like genogroup and the sea louse *C. rogercresseyi*.

Table S1. Primary polyclonal antibodies used in ELISA analysis.

Molecule	Source	Dilution	Reference
anti-Igs	Mouse	1:1,000	Supplementary Figure S1
anti-TNFα	Mouse	1:500	Morales-Lange et al. [1]
anti-IFNγ	Mouse	1:500	Morales-Lange et al. [1]

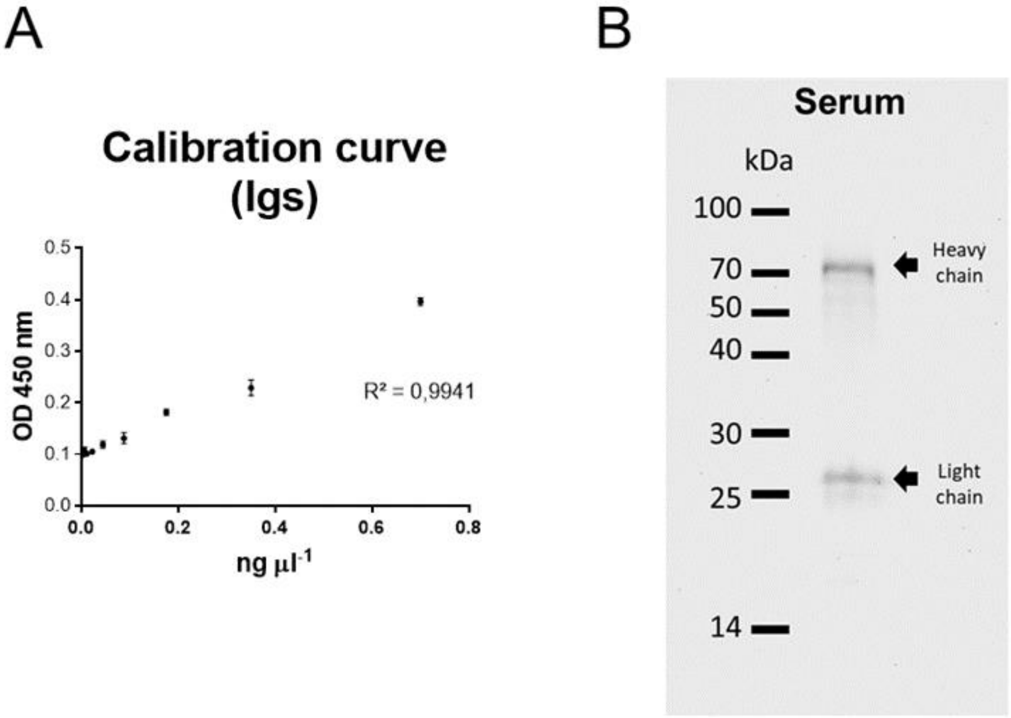

Figure S2. Validation of antibodies against total serum immunoglobulins (Igs) of *Salmo salar*: (**A**) Indirect ELISA calibration curve between total serum Igs concentration of *S. salar* (ng µL^{-1}) and optical density at 450 nm; (**B**) Western blot. Antibodies were produced in mice using total serum Igs from Atlantic salmon as antigen. The antigen was obtained by the caprylic acid technique for immunoglobulin purification [23].

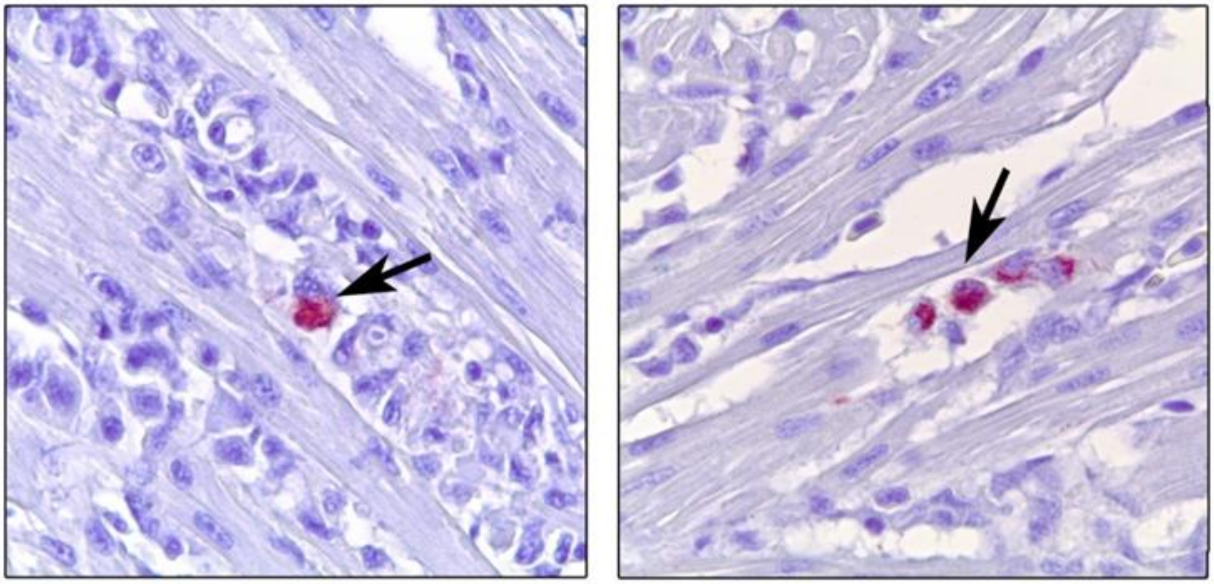

Figure S3. Presence of *Piscine orthoreovirus* (black arrows) in heart samples of Atlantic salmon from the first trial at day 41 post-infection with LF-89-like genogroup of *P. salmonis*. The virus was detected in 7 out of 17 fish analyzed by immunohistochemistry—magnification 63X.
